# Polymer Nanocomposites Containing Anisotropic Metal Nanostructures as Internal Strain Indicators

**DOI:** 10.3390/ma3021461

**Published:** 2010-02-24

**Authors:** Marco Bernabò, Andrea Pucci, Hasina Harimino Ramanitra, Giacomo Ruggeri

**Affiliations:** 1 Dipartimento di Chimica e Chimica Industriale, Università di Pisa, Via Risorgimento 35, 56126 Pisa, Italy; E-Mails: bernabo@ns.dcci.unipi.it (M.B.); harimino@libero.it (H.H.R.); grugge@dcci.unipi.it (G.R.); 2 CNR-INFM PolyLab c/o Dipartimento di Chimica e Chimica Industriale, Università di Pisa, via Risorgimento 35, I-56126 Pisa, Italy; 3 INSTM, c/o DCCI, Università di Pisa, via Risorgimento 35, I-56126 Pisa, Italy

**Keywords:** silver nanorods and nanowires, polymer nanocomposite films, dichroic properties

## Abstract

Polymer/metal nanocomposite containing intrinsically anisotropic metal nanostructures such as metal nanorods and nanowires appeared extremely more sensitive and responsive to mechanical stimuli than nanocomposites containing spherical nanoparticles. After uniaxial stretching of the supporting polymer matrix (poly(vinyl alcohol)), the elongated silver nanostructures embedded at low concentration into the polymer matrix (<1 wt % of Ag) assume the direction of the drawing, yielding materials with a strong dichroic response of the absorption behavior. Accordingly, the film changed its color when observed under linearly polarized light already at moderate drawings. The results obtained suggest that nanocomposite films have potential in applications such as color polarizing filters, radiation responsive polymeric objects and smart flexible films in packaging applications.

## 1. Introduction

Since the birth of nanotechnology, it was evident that the optical properties of nanostructured metal particles strongly depend on their dimensions and shape [1,2]. The absorption of visible light by metal nanoparticles was attributed to the induction of a collective oscillation of the free conduction electrons promoted by their interaction with electromagnetic field. Indeed, when the wavelength of an incident radiation is comparable with the mean free path of the conduction electrons of a metal particle, the electric component of the electromagnetic incident field induces the polarization of the conduction electrons, giving rise to the surface plasmon absorption [3].

Nowadays, there is a continued interest towards the optical properties of elongated noble metal nanostructures since their absorption spectrum is characterized by both a longitudinal and a transverse surface plasmon resonance, instead of only one as it happens for spherical nanoparticles [4,5,6,7,8]. Spherical particles, characterized by the highest symmetry, have only one plasmon resonance (all the resonance modes are degenerate), whereas by uniaxially extending a particle a second lower energy resonance band in the longitudinal direction takes place while the original plasmon resonance persists unaltered. In general, the number of surface plasmon resonance peaks increases as the particles symmetry decreases. One-dimensional structures longer than some hundreds nanometers up until micrometers or having a high aspect ratio are generally called nanowires, while smaller elongated structures are called nanorods.

Many techniques have been developed for the preparation of elongated nanostructures; in general, for the formation of nanorods and nanowires, anisotropic growth is required - meaning the crystal grows faster in only one direction [9,10]. One-dimensional systems can be actually obtained if the crystal growth proceeds mainly along a crystallographic axis, and there are several reasons to explain this process: different growth rate of the crystal facets, presence of imperfections in specific crystal directions such as screw dislocation, or the preferential accumulation of impurities on specific facets.

Interestingly, the optical properties of elongated nanostructures can be transferred to polymer matrices, thus yielding nanocomposite films with potentially responsive optical behavior towards external stimuli. It has been extensively demonstrated that uniaxial drawing of the polymer matrix allows embedded metal nanostructures to orient along the stretching direction, providing oriented films with absorption maximum depending on the polarization of the incident light [11,12,13,14]. As a consequence, if the embedded metal nanostructures are intrinsically anisotropic in shape, the overall dichroic absorption response of the nanocomposite should be more pronounced than that conferred by dispersed spherical metal nanoparticles.

In connection with these findings, we report in this work the preparation and the use of silver nanorods and nanowires as one-dimensional nanostructured metal probes for the detection of polymer matrix uniaxial deformation. In particular, both silver elongated nanostructures were prepared in solution by using a controlled solution-phase approach and successively dispersed into a water soluble poly(vinyl alcohol) (PVA) as polymer matrix. The nanocomposite films obtained after solvent evaporation were characterized by electronic microscopy (SEM and TEM) and X-ray diffraction in order to analyze the dispersion degree of the embedded metal nanostructures and the dichroic behavior provided by the anisotropic metal assemblies after films stretching evaluated by UV-Vis spectroscopy in polarized light.

## 2. Results and Discussion

### 2.1. Silver Nanorods Synthesis

One of the most effective methods reported in the literature for the preparation of silver nanorods [15,16] consists of a seed-mediated process using hexadecyltrimethylammonium bromide (CTAB) as surfactant and L-Ascorbic acid as reducing agent (see experimental part). Firstly, seed nanoparticles were obtained by the reduction of silver nitrate with a strong reducing agent such as sodium borohydride with the formation of small particles (typically ~9–12 nm) [15,17] according to the reaction Scheme 1:

(1) $$\text{AgN}{\text{O}}_{3}+\text{NaB}{\text{H}}_{4}\overset{{\text{H}}_{2}\text{O}}{\to }{\text{Ag}}^{0}+\frac{1}{2}{\text{H}}_{2}+\frac{1}{2}{\text{B}}_{2}{\text{H}}_{6}+\text{NaN}{\text{O}}_{3}$$

Successively the silver seed suspension was added to an alkaline water solution (*p*H ~10) containing additional silver salt, the surfactant (CTAB) and the reducing agent (ascorbic acid).

The silver reduction took place only after the addition of the seed: in fact, the L-Ascorbic acid (C_6_H_8_O_6_) is a weak reducing agent and it can reduce the Ag^+^ ions only in presence of a small amount of Ag(0) via a self-catalyzed process (Scheme 2).

(2) $${\text{C}}_{6}{\text{H}}_{8}{\text{O}}_{6}+2\text{AgN}{\text{O}}_{3}\overset{\text{Ag}(0)}{\to }2{\text{Ag}}^{0}+{\text{C}}_{8}{\text{H}}_{6}{\text{O}}_{6}+2\text{HN}{\text{O}}_{3}$$

**Figure 1:**
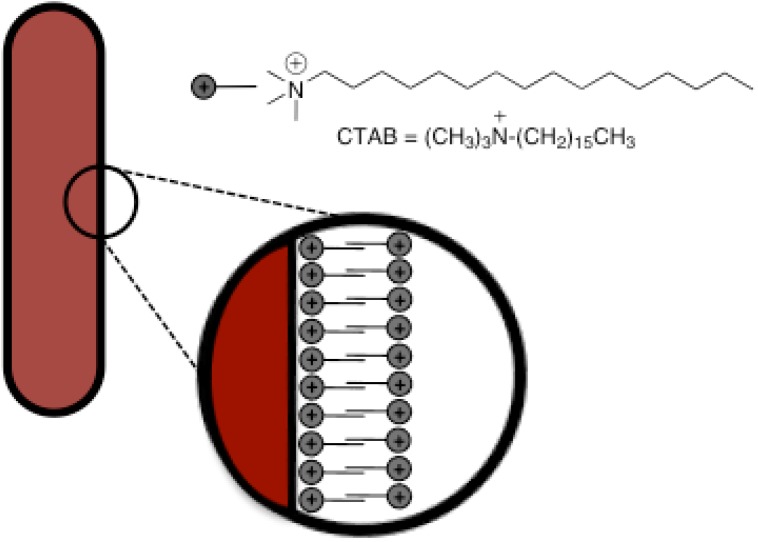
Schema of silver nanorod stabilization by CTAB capping.

In the seed-mediated process, the formation of nanorods is based on the preferential absorption of the surfactant (CTAB) to certain crystal facets of silver seed particles. In other words, CTAB binds to the surface radially but not axially, thus blocking crystal growth on these surfaces and allowing particle elongation in the axial direction only (Figure 1). [18]

**Figure 2:**
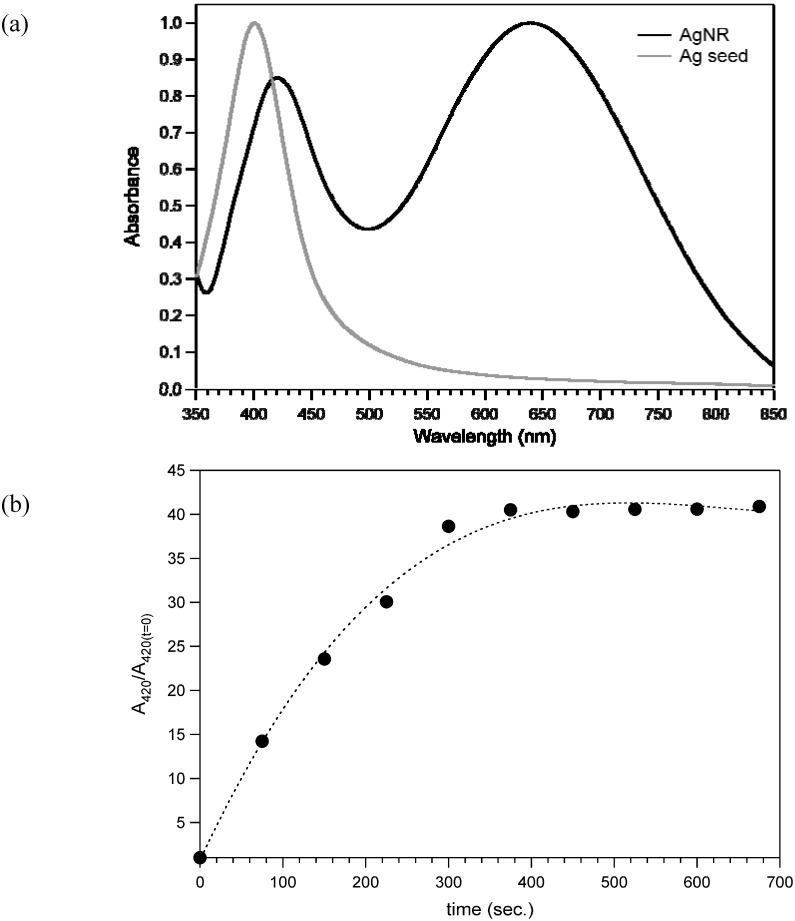
(a) UV-Vis absorption spectra of silver seed (grey) and nanorods (black) water dispersions. (b) Plot of the nanorods absorbance at 420 nm as a function of the reaction time with reference to the absorbance recorded at t = 0.

During this second step, a progressive color change took place due to particle growth; the initial yellow seed solution became reddish and finally green-grey. In order to confirm the anisotropic particle growth, the nanorod solution was analyzed by UV-Vis absorption spectroscopy and the spectrum was compared with the absorption of the pristine silver seed solution. The silver seed suspension showed only an absorption maximum at 410 nm while the rod solution displayed two resonances at 420 and 640 nm (Figure 2a).

In contrast to spherical nanoparticles, silver nanorods show actually two isolated plasmon absorption peaks, one at a 420 nm corresponding to the absorption and scattering of light along the short axis of the nanorod (transverse plasmon band), and the second at a longer wavelengths (640 nm) corresponding to the longitudinal plasmon resonance occurring along the long axis of the nanorod. [18]

The progressive increasing of the ratio At/At=0 at λ = 420 nm in the first steps of the reaction is likely related to morphological changes that occur to the seed particles (Figure 2a). After about six minutes, a plateau is reached, indicating that the structural changes of silver assemblies dispersed in solution were mostly over.

**Figure 3:**
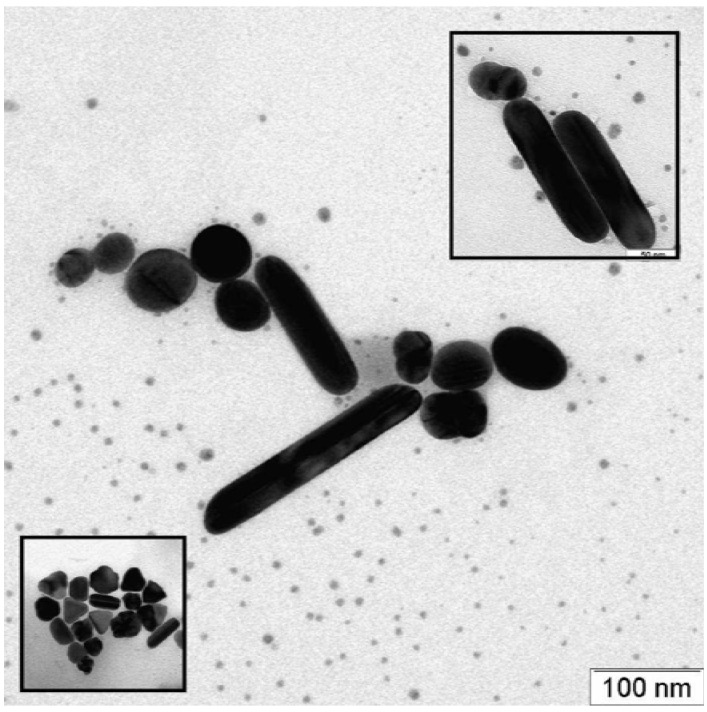
TEM micrograph of silver nanorods suspension after three min of reaction. Spherical and polygonal architectures like prisms and other geometrical features were present in the mixture as well (picture inset on the bottom left).

In order to confirm that the process gave rise to the formation of silver nanorods, the sample taken after about three min of reaction was characterized by transmission electron microscopy (TEM) analysis. The smallest silver nanoparticles and the surfactant excess that was present the reaction solution were removed by several washes with water, and the nanorods recovered by centrifugation at 3000 rpm for five minutes. Once washed, the rods were deposited onto the sample holder and then analyzed. The micrographs of the rods solution are shown in Figure 3: well-elongated structures with dimension between 150 and 225 nm (aspect ratio ~5–10) that were already formed after three minutes of reaction, but spherical and polygonal architectures like prisms and other geometrical features were present in the mixture as well (inset in Figure 3). It is worth noting the darker lines due to the diffraction of the crystallographic axis of the silver structures that are well evident along the major axis of the nanorods. This confirms the hypothesis that the structures were actually grown along a crystallographic axis as a consequence of the inhibiting action played by the surfactant (CTAB).

### 2.2. Silver Nanorods PVA Nanocomposites

The presence of intrinsically anisotropic metal nanostructures may suggest their use as highly dichroic assemblies into polymers. Indeed, it is possible to excite the transversal or the longitudinal mode in a well separated way by irradiating a dispersion of well aligned nanorods with linearly polarized light. [19]

Accordingly, the nanorods were then added to a water solution containing poly(vinyl alcohol) (PVA) and a homogeneous film was then obtained by solution casting after solvent evaporation. The silver based nanocomposite film (0.5 wt % of Ag) was finally uniaxially stretched (Dr = 5) at 110 °C in order to induce the dispersed nanorods to assume a preferential orientation along the drawing direction.

**Figure 4:**
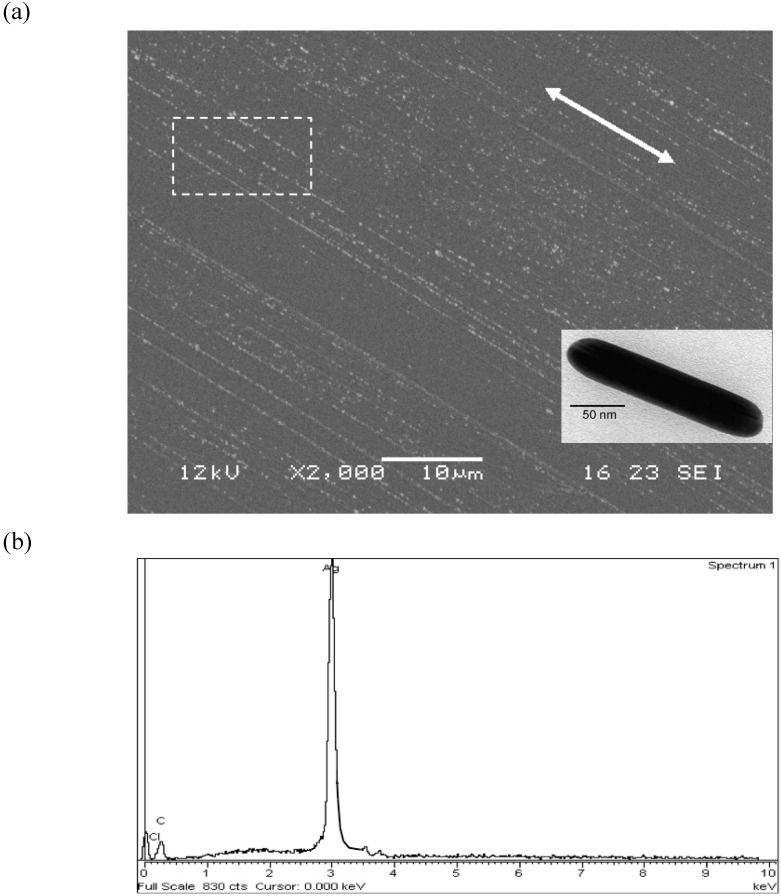
(a) SEM micrograph of a PVA@AgNR oriented film (Dr = 5) The white arrow denotes the stretching direction. In the inset: TEM image of an AgNR embedded in the polymer matrix. (b) Energy dispersive (EDS) spectrum of the film polymer surface. The white rectangle in (a) highlights the area where the EDS analysis was performed.

The morphology of the oriented portion of the nanocomposite was analyzed by SEM microscopy (Figure 4).

The micrograph reported in Figure 4 shows clearly the orientation of the silver assemblies along the stretching direction of the film (white arrow) and the rod-like shape of less than 50 nm of diameter of the single metal structure. Moreover, the energy dispersive (EDS) spectrum of the polymer film surface (Figure 4b) confirmed that these anisotropic objects are composed of Ag(0).

**Figure 5:**
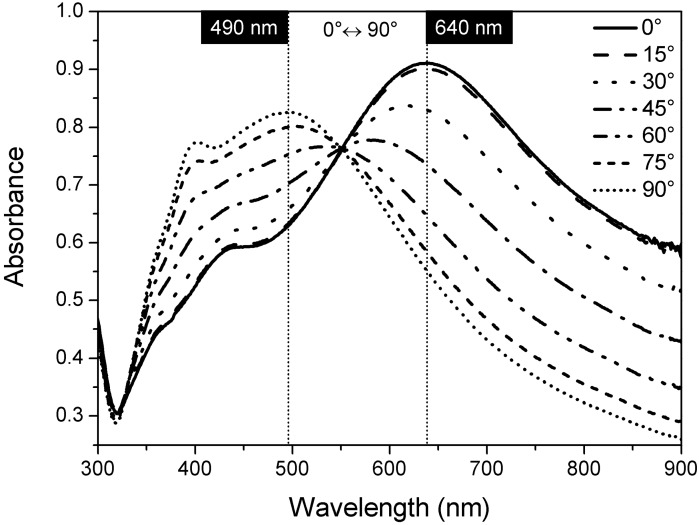
UV-Vis spectra of a PVA@AgNR oriented film (Dr = 5) as a function of the angle between the polarization light and the drawing direction.

The macroscopic dichroic behavior of the oriented PVA@AgNR film was evaluated by UV-Vis absorption spectroscopy in linearly polarized light (Figure 5).

It is worth noting that the variation from the parallel to the perpendicular relative orientation between the stretching direction of the film and the polarization vector of the incident light induced the strong decrease of the longitudinal mode of the surface plasmon resonance at 640 nm in favor of the transversal one at 420 nm, confirming the anisotropic distribution of the AgNR along the drawing orientation of the PVA matrix.

**Figure 6:**
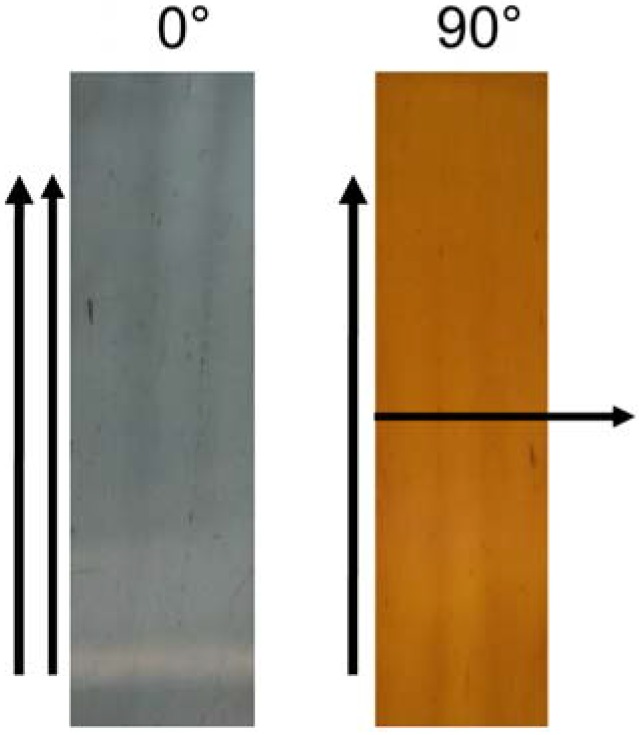
Images of oriented PVA@AgNR film (Dr = 5) observed through a linear polarizer with the transmission axis parallel (0°) and perpendicular (90°) to the drawing direction.

This dichroic behavior was also consistent with the digital images taken after the superimposition of a linear polarizer upon the oriented film (Figure 6).

The color of the film changed accordingly from blue-gray to reddish-brown simply by rotating the direction of the polarizer from the parallel configuration to the drawing axis (0°) to the perpendicular one (90°).

The large shift (~220 nm) in the wavelength of the two absorption maxima is also flanked by an increase of a red component placed at 500 nm with respect to the blue component located in the range from 600 to 900 nm.

**Figure 7:**
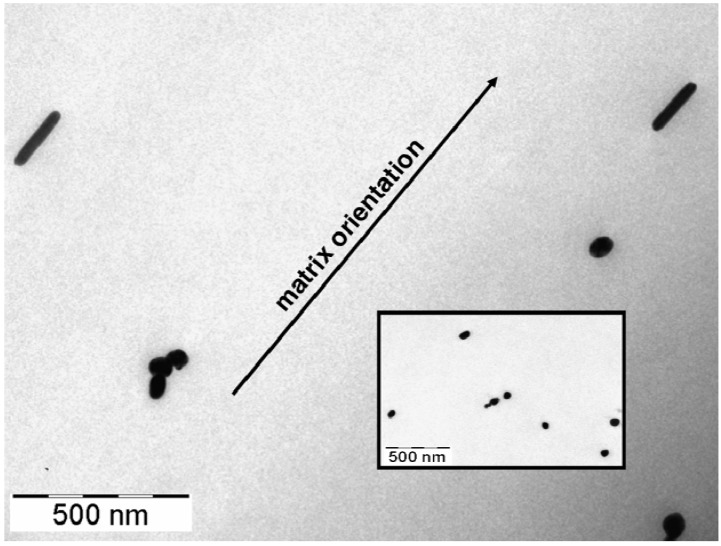
TEM micrograph of an oriented PVA@AgNR film (Dr = 5; the inset shows the presence of dispersed silver structures with dimensions between 50 and 100 nm).

In particular, the absorption band near 500 nm, absent in the nanorod dispersion, was probably due to the presence of silver structures with dimensions between 50 and 100 nm. These smaller particles could be likely generated during film preparation (casting and solvent evaporation) as a consequence of a sort of rearrangement and coalescence phenomena occurring to silver nanorods. Anyway, their absorption resulted in interestingly dichroic as well. It is likely that during the film preparation by solution casting, competition between the CTAB and the -OH groups of PVA could occur, thus promoting the surfactant desorption from the nanorod surface and the generation of isotropic more spherical silver nano-objects. This hypothesis was confirmed by the electron transmission microscopy (TEM) analysis of the stretched nanocomposite film: in Figure 7, the TEM micrograph displayed both the presence of nanoparticles of around 80–100 nm in size and the aligned nanorods.

In particular, the TEM micrograph displayed a concentration of silver objects embedded in the PVA matrix considerably less than that showed by SEM (Figure 4a). This behavior was attributed to the fact that TEM analysis investigated one of the inner layers of the PVA@AgNR film whereas SEM analyzed the overall sample surface. As already reported by us for analogously prepared gold or cadmium sulphide PVA nanocomposites by solution casting, [13,20] most of the metal-based assemblies were demonstrated to progressively accumulate close to the film surface exposed to air during solvent evaporation.

### 2.3. Silver Nanowires Synthesis

In the solution-phase approach used for the preparation of silver nanowires, silver nitrate was reduced in the presence of ethylene glycol (EG) at 160 °C. Then, silver nitrate and poly(vinyl pyrrolidone) (PVP) solutions were injected dropwise into the pre-heated EG dispersion, leading to the progressive generation of elongated nano-objects consisting of silver nanowires [21,22]. In particular, the formation of silver nanowires is based on the formation of preformed seeds from the initial reduction of AgNO_3_ in EG, which acts as both a reducing agent and disperding media. Indeed, due to the temperature-dependent reducing power of polyols (e.g., EG), they allow the control of both nucleation and growth processes through careful regulation of the reaction temperature. In addition, PVP helps to control the growth of crystalline faces, thus its concentration is critical to morphology. For example, PVP concentrations too high can induce isotropic growth, but also the order in which PVP and AgNO_3_ solutions are added plays a fundamental role for the formation of long silver nanowires.

**Figure 8:**
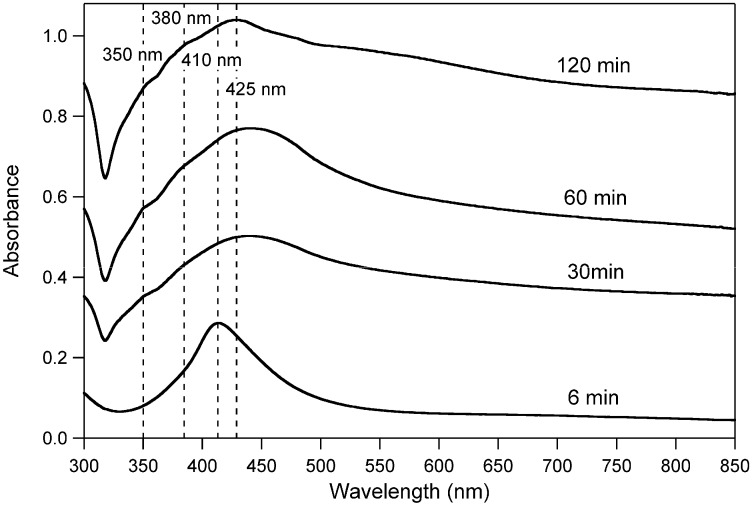
UV-Vis absorption spectra of the reaction mixture at various reaction times (min).

The formation of silver nanowires was monitored by UV-Vis absorption spectroscopy as a function of the reaction time (Figure 8).

In the first stages of the reaction, only the plasmon peak at 410 nm was visible and attributed to the formation of silver nanoparticles. As already reported in the literature [23], the broad full-width at half maximum of this band clearly indicates the presence of a broad distribution in size of the nanoparticles.

When the reaction had proceeded for more than 30 min, some new resonances appeared in the spectrum, together with a remarkable tailing effect attributed to the formation of particles with different morphologies (generally polyhedra and truncated nanocubes with large size) [22]. In particular, the two shoulders at respectively 350 and 380 nm indicating the formation of elongated silver nanostructures are attributed to the quadrupole resonance excitation of nanowires with different lengths [24]. The main peak 425 nm, which progressively evolved with time, was instead attributed to the transverse plasmon resonance of nanowires [21].

Moreover, according to the equation 3, [21]

λ_max_ = 361.3 + 0.410 D_nanowire_ (3)

which correlates the wavelength of the absorption maximum and the diameter of nanowires, the as-prepared elongated silver assemblies showed an average diameter of about 150 nm.

Considering all the optical features that characterize the absorption curve obtained after 120 minutes of reaction, the system is supposed to be formed by a mixture of silver nanowires and colloidal particles with different morphologies. In the purification step, consisting of dilution with acetone and centrifugation at 4000 rpm for about 30 min, the smaller silver nanoparticles were mostly removed from the crude mixture.

**Figure 9:**
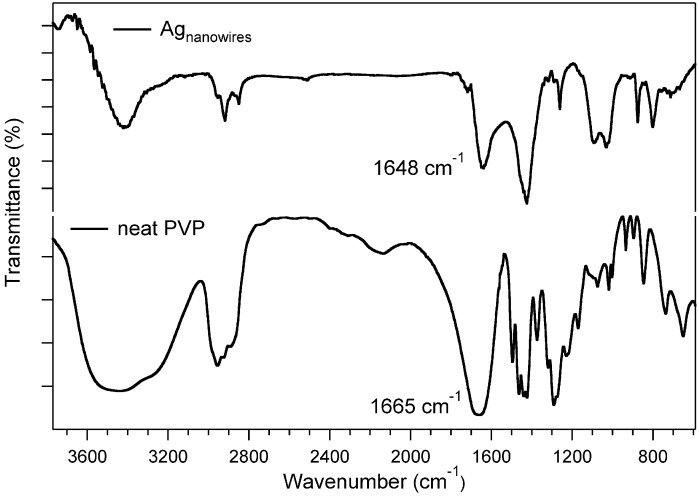
FT-IR spectra of neat PVP and silver nanowires.

In order to gain more information on the chemical nature of the silver nanowire – PVP interaction FT-IR investigations were carried out (Figure 9).

As reported by XPS investigations [25], the carbonyl group of PVP plays a pivotal role in the silver nanostructure stabilization. Accordingly, the FT-IR spectrum of silver nanowires showed all the signals attributed to the PVP matrix (3500, 2950, 1650, 1420, and 1295 cm^-1^; respectively assigned to the O–H stretching vibration (due to the hydrophilic nature of PVP), CH_2_ unsymmetrical, stretching vibration, C=O stretching vibration, CH_2_ bending vibration and C–N stretching vibration band) shift to lower energies of about 15 cm^-1^ of the carbonyl band with respect to neat PVP. This shift, attributed to a decrease of the double bond electron density, clearly indicates a strong mutual interaction between the metallic core and the protecting PVP chain.

The amount of the organic layer covering the purified silver nanostructures was determined by thermogravimetric analysis. The degradation curve showed a first weight loss from 100 to 250 °C of about 3.50% attributed to ethylene glycol desorption and a second from 400 to 600 °C of about 5.90% corresponding to PVP degradation. The silver nanowires were composed of globally 9.40 wt.% of organic stabilizing layer.

### 2.4. Silver Nanowires PVA Nanocomposites

As analogously reported for PVA@AgNR nanocomposites, the nanowires were then added to a water solution containing poly(vinyl alcohol) and a homogeneous film containing the 0.8 wt % of silver was then obtained by solution casting and solvent evaporation.

**Figure 10:**
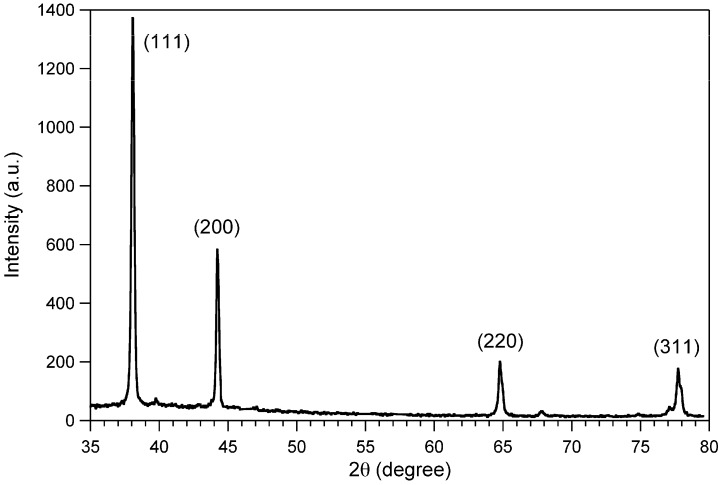
XRD pattern of PVA@AgNW film.

The presence of silver metal nanostructures embedded in the PVA matrix was confirmed by X-ray diffraction (XRD, Figure 10) analysis. The XRD pattern taken on PVA@AgNW film suggested that silver nanowires synthesized by using the solution-phase method existed purely in the face-center-cubic geometry (2θ = 38.1°, 44.3°, 64.5°, 77.4° for planes 111, 200, 220 and 311, respectively).

Moreover, it is worth noting the significant ratio between the intensity of the (111) and (200) peaks, which clearly indicates the enrichment of the (111) crystalline planes in the prepared silver nanowires.

**Figure 11:**
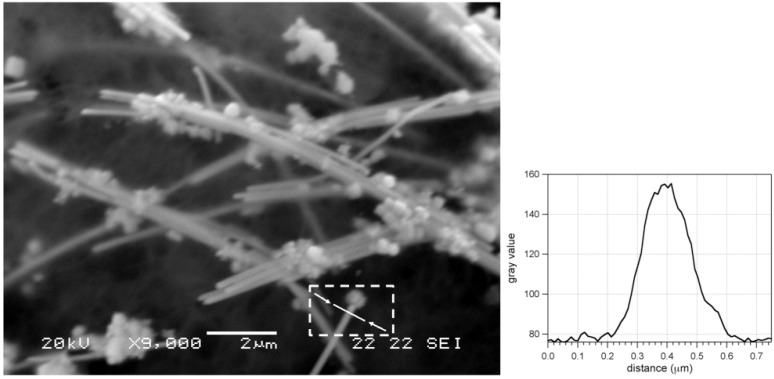
SEM micrograph of a PVA@AgNW film and plot profile of a silver nanowire (line scan within the white arrows).

The morphology of the PVA@AgNW film was then investigated by SEM. The micrograph, (Figure 11) revealed the presence of well-defined elongated silver nanostructures (~15 μm) characterized by a section diameter of about 160 nm (aspect ratio ≥90), consistent with the estimation provided by UV-Vis experiments (*i.e.*, 150 μm).

The embedded nanowires appeared well covered by a layer of the polymeric stabilizer and they were also in contact with more symmetric and spherical metal objects. These silver microstructures could likely have been formed during the production of nanowires and remained in the crude mixture even after purification, or they could have been generated as a consequence of the solution-casting process. As already reported for silver nanorods, a competition between the PVP stabilizing matrix and the -OH groups of PVA could occur during the film preparation, thus promoting the PVP desorption from the nanowire surface and the generation of more spherical silver agglomerates.

**Figure 12:**
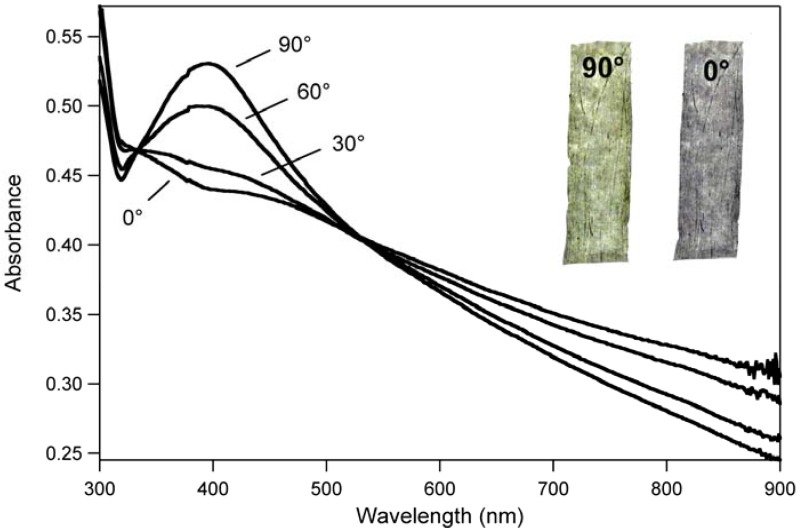
UV-Vis absorption spectra of an oriented PVA@AgNW film as a function of the angle between the polarization of light and the drawing direction of the ﬁlm, and images of the same film observed through a linear polarizer with the transmission axis parallel (0°) and perpendicular (90°) to the drawing direction (inset).

Preliminary interesting results were obtained after uniaxial orientation (Dr = 5) of the composite PVA@AgNW film at 110 °C. As reported by UV-Vis absorption experiments in polarized light as in Figure 12, the mechanical drawing induced to the film a pronounced dichroic behavior.

The main absorption of the oriented PVA@AgNW film pointed at about 390 nm mostly reduced its intensity when the polarization direction of the incident light shifted from the perpendicular (90°) to the parallel (0°) configuration with respect to the drawing axis. Most likely, the anisotropic behavior was attributed to the orientation of silver nanowires and larger metal aggregates embedded in the film towards the stretching direction. As a matter of fact, the main absorption at 380–400 nm, attributed to the transverse plasmon resonance of nanowires, strongly reduced its contribution when the polarization direction of the incident light was parallel (0°) to the PVA matrix orientation. Conversely, the absorption intensities in the near-infrared portion of the spectrum (700–900 nm) were higher in the parallel (0°) configuration with respect to the perpendicular one (90°), probably because in this region the dichoric behavior is primarily caused by the orientation of silver nanoparticles with different morphologies.

Those absorption features effectively conferred drawn PVA@AgNW films a strongly polarization-dependent color: *i.e.*, the color of the transmitted light through the oriented nanocomposite shifted from blue-gray to yellow-green when the angle between the polarization axis of an interposed polarizer and the drawing direction was varied from 0° to 90° (inset Figure 12).

Finally, the existence of well-defined isosbestic points (at 333 and 533 nm) effectively conﬁrmed the existence of two different populations of absorbing silver nanostructures. [12]

## 3. Experimental Section

### 3.1. Materials

Poly(vinyl alcohol) (PVA, 99+ % hydrolyzed, Mw = 146,000 − 186,000), poly(vinyl pyrrolidone) (PVP, average Mw ~55,000). All the other chemicals were purchased from Aldrich and were used without further purification. For the preparation of silver nanorods and nanowires, deionized and distilled water was used.

### 3.2. Sample Nomenclature

The samples were named by listing the polymer, the metal and the type of nanostructure, e.g., PVA@AgNR and PVA@AgNW are respectively the nanocomposites prepared by dispersing the two types of metal assemblies prepared in PVA.

### 3.3. Silver Nanorods Preparation *[15]*

10 mL of a solution containing 0.25 mM of AgNO_3_ and 0.25 mM of sodium citrate in water was reduced at room temperature and under vigorous stirring with 200 μL of 10 mM NaBH_4_ solution. After 30 seconds the stirring was stopped thus obtaining the silver seed solution.

10 mL of a 80 mM solution of hexadecyltrimethylammonium bromide (CTAB) containing 250 μL of 10 mM silver nitrate and 500 μL of 100 mM L- ascorbic acid in water were added to 60 μL of the fresh silver seed solution. Finally 100 μL of 1 M NaOH was added to obtain the desired nanorods. Stirring was not required, the solution was only gently shaken in order to have a complete mixing of NaOH within the solution.

The solution containing the silver nanorods was purified from the excess of surfactant (CTAB) by washing and recovery after several cycles of centrifugation at 3000 rpm.

### 3.4. Silver Nanowires Preparation *[21,25]*

2 mL of ethylene glycol (EG) was refluxed in flask at 180 °C for 30 min and then 1 mL of a 2 × 10^-4^ M solution of AgNO_3_ in EG was added. After 20 min of reaction, 5 mL EG solution of AgNO_3_ (0.1 M) and 5 ml EG solution of PVP (0.3 M) was simultaneously injected dropwise by syringe for 5 min. The reaction was maintained for a further 2 h. After cooling the as-obtained products were diluted with 110 mL of acetone and centrifuged at 4600 rpm for about 30 min. The supernatant, mainly composed of silver nanoparticles, was removed by syringe and the same washing operation was repeated until the supernatant phase appeared clear. Finally, ethanol was added to the product to dissolve the residual unbound PVP. After centrifugation, the supernatant was also removed by syringe, and the purified material preserved in ethanol.

### 3.5. Nanocomposite Preparation

200 mg of PVA was dissolved in 15 mL of water, with vigorous stirring at room temperature, and successively a water suspension of Ag nanorods or nanowires was added at a concentration that allowed the preparation of composite films with absorbances below 1 (*i.e.*, 0.8 wt %). Solid film was obtained by pouring the solution in a PTFE Petri dish until water evaporation (two days under a hood at room temperature).

The Ag/PVA nanocomposite films were successively stretched by uniaxial tensile drawing on a thermostatically controlled hot stage at a constant temperature of 120 °C at draw ratios (D_R_, defined as the ratio between the final and the initial length of the sample respectively) higher than 3.

### 3.6. Physical-Chemical Characterization

Infrared spectra were performed with a Fourier transform spectrometer “PerkinElmer™ Spectrum One” on KBr dispersions.

X-ray diffraction (XRD) patterns were obtained in Bragg-Brentano geometry with a Siemens D500 KRISTALLOFLEX 810 (CT: 1.0 s; SS: 0.050 dg and CuKα, λ = 1.541 Å) diffractometer. Data were acquired at room temperature.

Morphological and elemental characterization of nanocomposites were performed with a Carl Zeiss CEM-902 transmission electron microscope (TEM) equipped with a Castaing-Henry-Ottesmeyer energy filter spectrometer within the column. Solid polymeric films were analyzed by embedding each sample into Eponate812TM resin with BMDA as accelerator and leaving under air the embedded samples at 60 °C for 24 hours. Finally the embedded films were cut with the help of a Leica Reichert Ultracut obtaining thin cuts of about 60 nm thickness that were collected on a gold sample holder. The scanning electron microscopy (SEM) analysis was performed with a Jeol 5600-LV microscope, equipped with Oxford X-rays EDS microprobe.

Thermogravimetric scans were obtained with a Mettler Toledo Starc System moduloTGA/SDTA851^e^ under nitrogen flux, at a scan rate of 20 °C/min.

Particle analysis was performed using the public domain Image Tool 3.00 version image analyzer program developed at the University of Texas Health Science Center in San Antonio and available on Internet at http://ddsdx.uthscsa.edu/dig/itdesc.html.

UV-Vis absorption spectra of polymer films were recorded under isotropic conditions with a Perkin-Elmer Lambda 650, and in linearly polarized light by mounting motor-driven Glan-Taylor linear polarizers.

The film roughness was diminished using ultra-pure silicon oil [(poly(methyl phenylsiloxane)], 710® fluid, Aldrich) to reduce surface scattering between the polymeric films and the quartz slides used to keep them planar.

## 4. Conclusions

Elongated silver metal nanostructures such as nanorods and nanowires were synthesized in solution according to the controlled-shape methods starting from silver seed nanoparticle dispersions. Silver nanorods showed aspect ratios (*i.e.*, the length/diameter ratio of the object) of about 5–10 with an average length of about 200 nm, whereas silver nanowires were found to have aspect ratio of more than 90 and reached lengths of about 15–20 microns.

Once dispersed at moderate concentration (less than 1 wt % of Ag) into a PVA supporting matrix, most of the embedded silver nanorods or nanowires maintained their aspect and shape features, even if the competition of the –OH groups composing PVA towards silver nano-object stabilizers (CTAB for nanorods and PVP for nanowires) seemed to promote the formation of metal aggregates with larger size.

Interestingly, the resulting elongated silver assemblies followed the drawing direction of the supporting PVA matrix, thus yielding to extremely dichroic nanocomposite films that showed pronounced polarization-dependent absorption properties. The striking variation of the color of the mechanically stimulated films under polarization conditions suggests potential application of the nanocomposites as innovative smart flexible films in packaging applications.

## References

[1] Klabunde K.J. (2001). Nanoscale Materials in Chemistry.

[2] Kreibig U., Genzel L. (1985). Optical absorption of small metallic particles. Surf. Sci..

[3] Schwartzberg A.M., Zhang J.Z. (2008). Novel optical properties and emerging applications of metal nanostructures. J. Phys. Chem. C.

[4] Perez-Juste J., Rodriguez-Gonzalez B., Mulvaney P., Liz-Marzan L.M. (2005). Optical control and patterning of gold-nanorod-poly(vinyl alcohol) nanocomposite films. Adv. Funct. Mater..

[5] Mohamed M.B., Volkov V., Link S., El-Sayed M.A. (2000). The 'lightning' gold nanorods: Fluorescence enhancement of over a million compared to the gold metal. Chem. Phys. Lett..

[6] Maier S.A., Kik P.G., Atwater H.A., Meltzer S., Harel E., Koel B.E., Requicha A.A.G. (2003). Local detection of electromagnetic energy transport below the diffraction limit in metal nanoparticle plasmon waveguides. Nat. Mater..

[7] Jain P.K., Huang X., El-Sayed I.H., El-Sayed M.A. (2008). Noble metals on the nanoscale: Optical and photothermal properties and some applications in imaging, sensing, biology, and medicine. Acc. Chem. Res..

[8] Gluodenis M., Foss C.A. (2002). The effect of mutual orientation on the spectra of metal nanoparticle rod-rod and rod-sphere pairs. J. Phys. Chem. B.

[9] Ozin G.A., Arsenault A.C. (2005). Nanochemistry: A Chemical Approach to Nanomaterials.

[10] Cao G. (2004). Nanostructures and Nanomaterials, Synthesis, Properties and Applications.

[11] Caseri W. (2000). Nanocomposites of polymers and metals or semiconductors: Historical background and optical properties. Macromol. Rapid Commun..

[12] Bernabo M., Pucci A., Galembeck F., de Paula Leite C.A., Ruggeri G. (2009). Thermal- and sun-promoted generation of silver nanoparticles embedded into poly(vinyl alcohol) films. Macromol. Mater. Eng..

[13] Pucci A., Bernabò M., Elvati P., Meza L.I., Galembeck F., de Paula Leite C.A., Tirelli N., Ruggeri G. (2006). Photoinduced formation of gold nanoparticles into vinyl alcohol based polymers. J. Mater. Chem..

[14] Pucci A., Tirelli N., Willneff E.A., Schroeder S.L.M., Galembeck F., Ruggeri G. (2004). Evidence and use of metal-chromophore interactions: luminescence dichroism of terthiophene-coated gold nanoparticles in polyethylene oriented films. J. Mater. Chem..

[15] Jana N.R., Gearheart L., Murphy C.J. (2001). Wet chemical synthesis of silver nanorods and nanowires of controllable aspect ratio. Chem. Commun..

[16] Tao A.R., Habas S., Yang P. (2008). Shape control of colloidal metal nanocrystals. Small.

[17] Solomon S.D., Bahadory M., Jeyarajasingam A.V., Rutkowsky S.A., Boritz C., Mulfinger L. (2007). Synthesis and study of silver nanoparticles. J. Chem. Educ..

[18] Murphy C.J., Sau T.K., Gole A.M., Orendorff C.J., Gao J., Gou L., Hunyadi S.E., Li T. (2005). Anisotropic metal nanoparticles: Synthesis, assembly, and optical applications. J. Phys.Chem. B.

[19] Liz-Marzan L.M. (2006). Tailoring surface plasmons through the morphology and assembly of metal nanoparticles. Langmuir.

[20] Pucci A., Boccia M., Galembeck F., Leite C.A.D.P., Tirelli N., Ruggeri G. (2008). Luminescent nanocomposites containing CdS nanoparticles dispersed into vinyl alcohol based polymers. React. Funct. Polym..

[21] Chen C., Wang L., Jiang G., Zhou J., Chen X., Yu H., Yang Q. (2006). Study on the synthesis of silver nanowires with adjustable diameters through the polyol process. Nanotechnology.

[22] Kan C.-X., Zhu J.-J., Zhu X.-G. (2008). Silver nanostructures with well-controlled shapes. Synthesis, characterization and growth mechanisms. J. Phys. D Appl. Phys..

[23] Sun Y., Yin Y., Mayers B.T., Herricks T., Xia Y. (2002). Uniform silver nanowires synthesis by reducing AgNO3 with ethylene glycol in the presence of seeds and poly(vinyl pyrrolidone). Chem. Mater..

[24] Wang Z., Liu J., Chen X., Wan J., Qian Y. (2005). A simple hydrothermal route to large-scale synthesis of uniform silver nanowires. Chem. Eur. J..

[25] Jiang P., Li S.-Y., Xie S.-S., Gao Y., Song L. (2004). Machinable long PVP-stabilized silver nanowires. Chem. Eur. J..

